# Reproductive aging-associated common genetic variants and the risk of breast cancer

**DOI:** 10.1186/bcr3155

**Published:** 2012-03-20

**Authors:** Chunyan He, Daniel I Chasman, Jill Dreyfus, Shih-Jen Hwang, Rikje Ruiter, Serena Sanna, Julie E Buring, Lindsay Fernández-Rhodes, Nora Franceschini, Susan E Hankinson, Albert Hofman, Kathryn L Lunetta, Giuseppe Palmieri, Eleonora Porcu, Fernando Rivadeneira, Lynda M Rose, Greta L Splansky, Lisette Stolk, André G Uitterlinden, Stephen J Chanock, Laura Crisponi, Ellen W Demerath, Joanne M Murabito, Paul M Ridker, Bruno H Stricker, David J Hunter

**Affiliations:** 1 Department of Public Health, Indiana University School of Medicine, 980 West Walnut Street, R3-C241, Indianapolis, IN 46202, USA; 2 Indiana University Melvin and Bren Simon Cancer Center, 535 Barnhill Drive, Indianapolis, IN 46202, USA; 3 Division of Preventive Medicine, Brigham and Women's Hospital, Harvard Medical School, 900 Commonwealth Avenue East, Boston, MA 02215, USA; 4 Division of Epidemiology and Community Health, School of Public Health, 1300 South Second Street, Suite 300, University of Minnesota, Minneapolis, MN 55454, USA; 5 Framingham Heart Study, The National Heart Lung and Blood Institute, 73 Mount Wayte, Suite 2, Framingham, MA 01701, USA; 6 Department of Epidemiology, Erasmus Medical Center, 3015 GE, Rotterdam, The Netherlands; 7 Istituto di Ricerca Genetica e Biomedica, Consiglio Nazionale delle Ricerche, c/o Cittadella Universitaria di Monserrato, Monserrato, 09042 Cagliari, Italy; 8 Department of Epidemiology, Gillings School of Global Public Health, University of North Carolina at Chapel Hill, 137 East Franklin Street, Suite 306, CB #8050, Chapel Hill, NC 27599, USA; 9 Department of Epidemiology, Harvard School of Public Health, 677 Huntington Avenue, Boston, MA 02115, USA; 10 Channing Laboratory, Department of Medicine, Brigham and Women's Hospital, and Harvard medical School, 181 Longwood Avenue, Boston, MA 02115, USA; 11 Department of Biostatistics, Boston University School of Public Health, 801 Massachusetts Avenue, 3rd floor, Boston, MA 02118, USA; 12 Unit of Cancer Genetics, Istituto di Chimica Biomolecolare, Consiglio Nazionale delle Ricerche, Li Punti, 07100 Sassari, Italy; 13 Department of Internal Medicine, Erasmus Medical Center, 3000 CA, Rotterdam, The Netherlands; 14 Netherlands Consortium of Healthy Aging, Rotterdam, Rotterdam, The Netherlands; 15 Division of Cancer Epidemiology and Genetics, National Cancer Institute, National Institutes of Health, Department of Health and Human Services, 6120 Executive Boulevard, Bethesda, MD 20892, USA; 16 Sections of General Internal Medicine, Department of Medicine, Boston University School of Medicine, 720 East Concord Street, Boston, MA 02118, USA; 17 Program in Medical and Population Genetics, Broad Institute of Harvard University and MIT, 301 Binney Street, Cambridge, MA 02142, USA

## Abstract

**Introduction:**

A younger age at menarche and an older age at menopause are well established risk factors for breast cancer. Recent genome-wide association studies have identified several novel genetic loci associated with these two traits. However, the association between these loci and breast cancer risk is unknown.

**Methods:**

In this study, we investigated 19 and 17 newly identified single nucleotide polymorphisms (SNPs) from the ReproGen Consortium that have been associated with age at menarche and age at natural menopause, respectively, and assessed their associations with breast cancer risk in 6 population-based studies among up to 3,683 breast cancer cases and 34,174 controls in white women of European ancestry. In addition, we used these SNPs to calculate genetic risk scores (GRSs) based on their associations with each trait.

**Results:**

After adjusting for age and potential population stratification, two age at menarche associated SNPs (rs1079866 and rs7821178) and one age at natural menopause associated SNP (rs2517388) were associated with breast cancer risk (p values, 0.003, 0.009 and 0.023, respectively). The odds ratios for breast cancer corresponding to per-risk-allele were 1.14 (95% CI, 1.05 to 1.24), 1.08 (95% CI, 1.02 to 1.15) and 1.10 (95% CI, 1.01 to 1.20), respectively, and were in the direction predicted by their associations with age at menarche or age at natural menopause. These associations did not appear to be attenuated by further controlling for self-reported age at menarche, age at natural menopause, or known breast cancer susceptibility loci. Although we did not observe a statistically significant association between any GRS for reproductive aging and breast cancer risk, the 4^th ^and 5^th ^highest quintiles of the younger age at menarche GRS had odds ratios of 1.14 (95% CI, 1.01 to 1.28) and 1.13 (95% CI, 1.00 to 1.27), respectively, compared to the lowest quintile.

**Conclusions:**

Our study suggests that three genetic variants, independent of their associations with age at menarche or age at natural menopause, were associated with breast cancer risk and may contribute modestly to breast cancer risk prediction; however, the combination of the 19 age at menarche or the 17 age at natural menopause associated SNPs did not appear to be useful for identifying a high risk subgroup for breast cancer.

## Introduction

A younger age at menarche and an older age at menopause are well-established risk factors for the development of breast cancer [1]. In the general population, the risk of breast cancer decreases by 10% for each 2-year delay in menarche [2] but increases by 3% for each year that menopause is delayed [3]. These associations are consistent with the hypothesis that breast cancer risk is related to the extent of steroid hormone exposure during a woman's reproductive years, which drives breast mitotic activity and determines the probability of tumorigenic somatic events [4].

Recently, genome-wide association studies (GWAS) have identified several new common genetic loci associated with either age at menarche or age at natural menopause. Four independent GWAS of age at menarche have identified two novel loci at *LIN28B *and 9q31.2 [5-8], and two GWAS of age at natural menopause have identified four novel loci on chromosomes 5, 6, 19, and 20 [5,9]. Most recently, the ReproGen Consortium, which consisted of these initial GWASs and many additional studies, has conducted expanded meta-analyses for age at menarche [10] and age at natural menopause [11] and reported more new loci identified for each trait. Given the well-established associations of age at menarche and age at natural menopause with breast cancer risk, we set out to assess whether these common genetic loci influence breast cancer risk and whether a genetic risk score (GRS) for these reproductive events might be useful for identifying a high-risk subgroup for breast cancer. Furthermore, since the reproductive risk factors have been observed to be differentially associated with breast cancer by tumor histological subtypes [12-16], we assessed these genetic associations by tumor histological subtypes defined by estrogen receptor (ER) status.

We therefore conducted a meta-analysis of six population-based studies to investigate the association between genetic loci associated with age at menarche or age at natural menopause and breast cancer risk. We assessed 19 and 17 single-nucleotide polymorphisms (SNPs) that have been previously reported to be linked to age at menarche [10] and age at natural menopause [11], respectively, among up to 3,683 breast cancer cases and 34,174 controls in women of European ancestry and evaluated whether these SNPs were differentially associated with breast cancer subtypes defined by ER status in two studies in which such data were available.

## Materials and methods

### Study population

The ReproGen Consortium was formed by more than 30 studies in the US and Europe to investigate the genetics of reproductive aging traits [10,11]. Our analysis used data from six population-based studies from the ReproGen Consortium: the Nurses' Health Study (NHS), the Women's Genome Health Study (WGHS), the SardiNIA Breast Cancer Study (SardiNIA), the Rotterdam Study I and II (RSI+II), the Framingham Heart Study (FHS), and the Atherosclerosis Risk in Communities Study (ARIC). Each study had at least 200 breast cancer cases. Four studies were prospective cohort studies, one was a nested case-control study, and one was a case-control study. A description of the six studies is provided in Table 1, and more information is given in Additional file 1. Briefly, breast cancer cases occurring in defined populations during specific periods of time were identified by structured questionnaires, medical records, or linkage with a nationwide registry of cancer or death index or both. By the time we conducted this study, the majority of the women in these studies had passed through menopause. As most of the participants in these studies were European whites, we restricted analyses to women of European ancestry. We excluded subjects with missing information on age. Two studies (NHS and WGHS) provided information on the ER status of the breast tumors for a subset of the cases. This information was extracted from medical records. Each study was approved by the relevant local institutional review boards.

**Table 1:** List of participating studies and number of case and control subjects

Study acronym	Study name	Study design	Case subjects (*n *= 3,683)	ER^+^/ER^- ^subjects (*n *= 1,716/371)	Control subjects (*n *= 34,174)	All subjects (*n *= 37,857)
NHS-BC	Nurses' Health Study-Breast Cancer	Nested case-control	1,145 (31.1)	807/181	1,142 (3.3)	2,287 (6.0)
WGHS	Women's Genome Health Study	Prospective cohort	1,099 (29.8)	909/190	22,205 (65.0)	23,304 (61.5)
SardBC	SardiNIA Breast Cancer Study	Case-control	809 (22.0)	-	674 (2.0)	1,483 (3.9)
RSI+II	Rotterdam Study I and II	Prospective cohort	216 (5.9)	-	4,261 (12.5)	4,477 (11.8)
FHS	Framingham Heart Study	Prospective cohort	207 (5.6)	-	3,698 (10.8)	3,905 (10.3)
ARIC	Atherosclerosis Risk in Communities Study	Prospective cohort	207 (5.6)	-	2,194 (6.4)	2,401 (6.3)

Data are presented as the number (percentage) of cases, controls, and all subjects. ER, estrogen receptor.

### Genotype data

We analyzed genotypes for 19 and 17 independent SNPs with reported associations with age at menarche and age at natural menopause, respectively, in the ReproGen Consortium, in which all SNPs achieved genome-wide significance in the meta-analysis of each trait (combined stage 1 and replication *P *value of less than 1 × 10^-8^) [10,11]. None of these SNPs has been reported to be associated with breast cancer risk in previous GWAS and this is likely because of the very stringent *P *value threshold used to declare genome-wide significance (usually, *P *values were less than at least 1 × 10^-7^). As positive controls, 10 SNPs with consistently reported associations with breast cancer as shown in recent GWAS were included [17-19]. All 46 SNPs are listed in Table S1 of Additional file 2. Genotypes used in this analysis have been previously described [10,11]. Complete genotype data from a total of up to 3,683 cases and 34,174 control subjects were available for analysis after the exclusions described in the 'Study population' section.

### Breast cancer risk factors

The six studies from the ReproGen Consortium provided information on one or more of the following risk factors for breast cancer: age (continuous, at study entry or diagnosis), age at menarche (continuous, between 9 and 17 years), age at natural menopause (continuous, between 40 and 60 years), age at first live birth (less than 20, 20 to 24, 25 to 29 or no birth, at least 30 years), family history of breast cancer in first-degree relatives (yes/no), alcohol consumption (less than 5, 5 to 15, 15 to 30, at least 30 g/day), parity (0, 1 to 2, at least 3), menopausal hormone therapy (ever/never), oral contraceptive (OC) use (ever/never), and adult body mass index (BMI) (continuous).

### Genetic risk score computation

The GRS was calculated on the basis of the 19 and 17 independent SNPs identified in previous studies as being associated with age at menarche and age at natural menopause, respectively [10,11]. As a younger age at menarche and an older age at menopause are independently associated with an elevated breast cancer risk, we computed separate GRSs for a younger age at menarche and an older age at natural menopause. The risk allele was defined as an allele that was associated with a younger age at menarche or an older age at natural menopause. Two methods were used to determine the GRS: a simple count method (count GRS) and a weighted method (weighted GRS). We assumed an additive genetic model for each SNP, applying a linear weighting of 0, 1, or 2 to genotypes containing 0, 1, or 2 risk alleles, respectively. The count method assumes that each SNP contributes equally to the risk of breast cancer. The count GRS was calculated by simply summing the number of risk alleles of each SNP. For the weighted GRS, each SNP was weighted by β-coefficients obtained from the replication studies of recent meta-analyses of two traits [10,11]. The weighted GRS was calculated by multiplying each β-coefficient by the number of corresponding risk alleles (0, 1, or 2) and then summing the products. To simplify interpretation and facilitate comparison with the count GRS, the weighted GRS was further divided by twice the sum of the β-coefficients and then multiplied by the total number of risk alleles. To provide a positive control and also to control for potential confounding by known breast cancer-associated genetic variants, a count GRS was computed on the basis of the 10 SNPs with consistently reported associations with breast cancer [19]: rs2981582, rs3803662, rs11249433, rs7716600, rs13387042, rs889312, rs13281615, rs999737, rs3817198, and rs1045485.

### Statistical analysis

In each of the six studies, we performed logistic regression to evaluate the association with breast cancer for each of the 46 candidate SNPs, assuming an additive genetic model. Logistic regression was also used to analyze the association between GRS and breast cancer by including both GRSs for age at menarche and age at natural menopause in the model as the main effects. The GRSs were modeled as continuous variables or categorized into quintiles, and the cutoff points for quintiles were based on the WGHS population, which is the largest prospective cohort population among all participating studies. This approach was applied to each of the six participating studies. Odds ratios (ORs) and 95% confidence intervals (CIs) were estimated from logistic regression. To control for potential confounding by population stratification, we adjusted for the top principal components of genetic variation chosen for each study. We adjusted for age in the main model. To examine whether the genetic association of each of the candidate SNPs or GRSs with breast cancer is mediated through the onset of menarche or natural menopause, we then adjusted for self-reported age at menarche and age at natural menopause in the main model. Other conventional risk factors for breast cancer - including age at first live birth, family history of breast cancer in first-degree relatives, alcohol consumption, parity, menopausal hormone therapy, OC use, and adult BMI - were further included in the model to control for potential confounding in studies which had such data available. To examine whether these genetic associations differ by breast cancer subtypes, in each of the two studies that provided information on ER status, we then investigated the genetic association of each of the candidate SNPs or GRSs with breast cancer in subgroup analysis by ER histological status (positive or negative).

Forest plots were used to present study-specific ORs and 95% CIs. We then performed meta-analyses by using the fixed-effects model to estimate summary ORs from study-specific estimatesthat were weighted by the inverse of the variance of each study. As the meta-analyses restricted to prospective cohort studies or case-control studies yielded similar results, we present results from only the meta-analysis of all six participating studies. We also tested the heterogeneity of associations across studies as well as across different tumor subtypes by using the Q test [20].

All statistical analyses were performed by using SAS version 9.1 software (SAS Institute Inc., Cary, NC, USA). Power calculations were carried out by using Quanto (University of Southern California, Los Angeles, CA, USA). All *P *values were based on two-sided tests and were considered statistically significant if less than 0.05. Because SNPs were selected on the basis of an *a priori *hypothesis, adjustments for multiple comparison tests were not performed.

## Results

The six participating studies contributed 3,683 breast cancer cases and 34,174 controls of self-reported white women of European ancestry (Table 1), all with available data on age and the 46 candidate SNPs, and at least one of the conventional risk factors considered. Of the 3,683 cases, about 52% were from the four prospective cohort studies (WGHS, RSI+II, FHS, and ARIC), about 30% were from the nested case-control study in NHS, and about 18% were from the population-based case-control study in SardiNIA. ER status was known for 2,087 cases in the NHS and the WGHS. On average, compared with the controls, the cases had a younger age at menarche and an older age at natural menopause. The expected associations with breast cancer were generally observed for the conventional risk factors across all of the studies (Table S2 of Additional file 3). The associations of the 46 candidate SNPs with age at menarche or age at natural menopause in the six studies were consistent with the original findings from the two meta-analyses [10,11].

**Figure 1:**
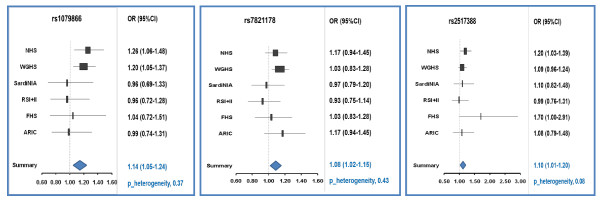
**Forest plots for the three candidate loci (rs1079866, rs7821178, and rs2517388) in association with breast cancer risk**. Per-risk-allele odds ratios (ORs) and 95% confidence intervals (CIs) were obtained from unconditional logistic regression in each study, and age and potential population stratification were adjusted for. The size of the box is inversely proportional to the standard error of the log OR estimate. *P *values for heterogeneity across studies are 0.37, 0.43, and 0.08, respectively. ARIC, Atherosclerosis Risk in Communities Study; FHS, Framingham Heart Study; NHS, Nurses' Health Study; RSI+II, Rotterdam Study I, II; SardiNIA, SardiNIA Breast Cancer Study; WGHS, Women's Genome Health Study.

Table 2 shows the risk allele frequency and the corresponding per-risk-allele OR of breast cancer for each of the 46 candidate SNPs. The results are arranged in order of the strength of statistical significance (*P *value). The allele frequency for each SNP in the controls was similar to those reported for populations of European descent [21-23]. After adjusting for age and potential population stratification, we found that, among the 19 candidate SNPs for a younger age at menarche, two SNPs, rs1079866 and rs7821178, were significantly associated with breast cancer risk and had corresponding per-risk-allele ORs of 1.14 (96% CI = 1.05 to 1.24; *P *value = 0.003; *P *for heterogeneity = 0.37) and 1.08 (95% CI = 1.02 to 1.15; *P *value = 0.009; *P *for heterogeneity = 0.43), respectively. The SNP rs1079866 is located about 250 kb away from the *INHBA *gene on chromosome 7, whereas SNP rs7821178 is about 181 kb away from the *PXMP3 *gene (also known as *PEX3*) on chromosome 8. The strongest GWAS hit for age at menarche, rs7759938 at *LIN28B *on chromosome 6, was not found to be associated with breast cancer risk (*P *value = 0.60). Of the 17 candidate SNPs associated with an older age at natural menopause, one SNP, rs2517388, was significantly associated with breast cancer risk with a per-risk-allele OR of 1.10 (95% CI = 1.01 to 1.20; *P *value = 0.023; *P *for heterogeneity = 0.08). This SNP is an intronic SNP in the *ASH2L *gene on chromosome 8. The study-specific and summary ORs for the three associated SNPs are shown in Figure 1. Further adjustment for conventional risk factors - including age at menarche, age at natural menopause, age at first live birth, family history of breast cancer in first-degree relatives, alcohol consumption, parity, menopausal hormone therapy, OC use, and adult BMI - did not change the results substantially. For candidate loci for age at menarche and age at natural menopause, the findings did not differ materially when we further adjusted for known breast cancer-associated SNPs.

**Table 2:** Association of candidate single-nucleotide polymorphism loci and the risk of breast cancer

SNP	Gene(s)^a^	Distance from gene	Chromosome	Position (Build 36)	Risk/Reference alleles^b^	Effect allele	Allele frequency^c ^of cases/controls^d^	Effect	SE	OR (95% CI)^e^	*P *value^f^	Direction^g^	*P *value for heterogeneity^h^
Age at menarche													
rs1079866	*INHBA*	~250 kb	7	41436618	C/G	c	0.87/0.85	0.132	0.044	1.14 (1.05-1.24)	0.003	++--+-	0.37
rs7821178	*PXMP3*	~181 kb	8	78256392	A/C	a	0.35/0.33	0.081	0.031	1.08 (1.02-1.15)	0.009	++--++	0.43
rs7642134	*VGLL3*	~70 kb	3	86999572	A/G	a	0.39/0.38	0.058	0.03	1.06 (1.00-1.12)	0.056	++++-+	0.83
rs10980926	*ZNF483*	Intronic	9	113333455	G/A	g	0.64/0.65	-0.057	0.031	0.94 (0.89-1.00)	0.066	---+--	0.58
rs1398217	*FUSSEL18*	Intronic	18	43006236	G/C	g	0.42/0.42	0.048	0.03	1.05 (0.99-1.11)	0.10	++-+++	0.40
rs17188434	*NR4A2*	~84 kb	2	156805022	C/T	c	0.07/0.07	0.075	0.059	1.08 (0.96-1.21)	0.20	+++---	0.34
rs13187289	*PHF15*	~12 kb	5	133877076	C/G	c	0.80/0.80	-0.038	0.038	0.96 (0.89-1.04)	0.32	---+-+	0.03
rs12617311	*PLCL1*	~195 kb	2	199340810	A/G	a	0.32/0.32	-0.032	0.033	0.97 (0.91-1.03)	0.33	--+-+-	0.81
rs17268785	*CCDC85A*	Intronic	2	56445587	A/G	a	0.84/0.84	0.037	0.041	1.04 (0.96-1.12)	0.37	-+-+++	0.89
rs2002675	*TRA2B, ETV5*	~4 kb, ~135 kb	3	187112262	A/G	a	0.58/0.57	0.022	0.03	1.02 (0.96-1.08)	0.47	++++--	0.92
rs466639	*RXRG*	Intronic	1	163661506	T/C	t	0.12/0.12	-0.032	0.046	0.97 (0.89-1.06)	0.49	-+-+--	0.35
rs1659127	*MKL2*	~28 kb	16	14295806	G/A	g	0.67/0.66	0.022	0.033	1.02 (0.96-1.09)	0.50	+++++-	0.28
rs9635759	*CA10*	~94 kb	17	46968784	G/A	g	0.69/0.70	-0.021	0.034	0.98 (0.92-1.05)	0.54	-+--+-	0.32
rs10899489	*GAB2*	Intronic	11	77773021	C/A	c	0.85/0.84	0.025	0.041	1.03 (0.95-1.11)	0.54	++++--	0.97
rs10423674	*CRTC1*	Intronic	19	18678903	C/A	c	0.67/0.67	0.017	0.032	1.02 (0.96-1.08)	0.59	+++-+-	0.60
rs7759938	*LIN28B*	~26 kb	6	105485647	T/C	t	0.69/0.69	-0.017	0.032	0.98 (0.92-1.05)	0.60	+-+---	0.53
rs2090409	*TMEM38B*	~400 kb	9	108006909	A/C	a	0.33/0.33	0.012	0.031	1.01 (0.95-1.08)	0.69	--++-+	0.21
rs6438424	*3q13.32*	Intergenic	3	119057512	A/C	a	0.50/0.50	0.002	0.029	1.00 (0.95-1.06)	0.94	---+++	0.88
rs6589964	*BSX*	~18 kb	11	122375893	A/C	a	0.48/0.48	-0.001	0.031	1.00 (0.94-1.06)	0.99	--++++	0.20
Age at natural menopause													
rs2517388	*ASH2L*	Intronic	8	38096889	G/T	g	0.17/0.16	0.096	0.042	1.10 (1.01-1.20)	0.023	+++-+-	0.08
rs4693089	*HEL308*	Intronic	4	84592646	G/A	g	0.47/0.48	-0.06	0.031	0.94 (0.89-1.00)	0.054	------	0.38
rs11668344	*TMEM150B*	Intronic	19	60525476	A/G	a	0.64/0.64	-0.049	0.03	0.95 (0.90-1.01)	0.11	+-----	0.02
rs2307449	*POLG*	Intronic	15	87664932	T/G	t	0.60/0.61	-0.045	0.031	0.96 (0.90-1.02)	0.15	-+--+-	0.20
rs16991615	*MCM8*	Missense	20	5896227	A/G	a	0.07/0.06	0.079	0.06	1.08 (0.96-1.22)	0.18	+++--+	0.80
rs12294104	*C11orf46, PPED2*	~24 kb,~49 kb	11	30339475	T/C	t	0.17/0.17	0.046	0.039	1.05 (0.97-1.13)	0.24	++-++-	0.56
rs12461110	*NLRP11*	Missense	19	61012475	G/A	g	0.64/0.65	-0.036	0.031	0.96 (0.91-1.03)	0.24	--++-+	0.32
rs2303369	*FNDC4*	Intronic	2	27568920	C/T	c	0.62/0.63	-0.031	0.03	0.97 (0.91-1.03)	0.31	--++-+	0.22
rs4886238	*TDRD3*	Intronic	13	60011740	A/G	a	0.35/0.34	0.029	0.031	1.03 (0.97-1.09)	0.35	++----	0.79
rs2277339	*PRIM1*	Missense	12	55432336	T/G	t	0.90/0.89	0.037	0.05	1.04 (0.94-1.14)	0.46	++++--	0.47
rs10852344	*GSPT1, TNFRSF17*	~7 kb, ~42 kb	16	11924420	C/T	c	0.41/0.41	0.020	0.030	1.02 (0.96-1.08)	0.50	++--++	0.87
rs10183486	*TLK1*	Intronic	2	171699217	C/T	c	0.62/0.62	-0.015	0.031	0.99 (0.93-1.05)	0.63	--+--+	0.98
rs4246511	*RHBDL2*	Intronic	1	39152972	T/C	t	0.27/0.27	0.016	0.035	1.02 (0.95-1.09)	0.66	++---+	0.69
rs2153157	*SYCP2L*	Intronic	6	11005474	A/G	a	0.50/0.50	0.011	0.029	1.01 (0.96-1.07)	0.70	++-+-+	0.59
rs1635501	*EXO1*	Intronic	1	240107398	T/C	t	0.53/0.52	0.011	0.034	1.01 (0.95-1.08)	0.74	+-+--+	0.31
rs365132	*UIMC1*	Synonymous	5	176311180	T/G	t	0.50/0.50	-0.009	0.03	0.99 (0.93-1.05)	0.76	+-+--+	0.58
rs1046089	*BAT2*	Missense	6	31710946	G/A	g	0.65/0.65	-0.008	0.031	0.99 (0.93-1.05)	0.79	-+-+-+	0.24
Breast cancer													
rs2981582	*FGFR2*	Intronic	10	123342307	A/G	a	0.44/0.38	0.188	0.03	1.21 (1.14-1.28)	4.7 × 10^-10^	++++++	0.10
rs3803662	*TOX3*	~6 kb	16	51143842	A/G	a	0.31/0.25	0.166	0.032	1.18 (1.11-1.26)	2.6 × 10^-7^	++++-+	0.31
rs11249433	*FCGR1B*	~245 kb	1	120982136	G/A	g	0.44/0.46	0.149	0.031	1.16 (1.09-1.23)	1.9 × 10^-6^	+++++-	0.26
rs7716600	*MRPS30*	~59 kb	5	44910762	A/C	a	0.24/0.20	0.148	0.035	1.16 (1.08-1.24)	2.3 × 10^-5^	++++++	0.80
rs13387042	*TNP1*	~181 kb	2	217614077	A/G	a	0.55/0.50	0.114	0.029	1.12 (1.06-1.19)	1.0 × 10^-4^	++-+++	0.20
rs889312	*MAP3K1*	~43 kb	5	56067641	C/A	c	0.30/0.32	0.122	0.032	1.13 (1.06-1.20)	1.5 × 10^-4^	++++++	0.65
rs13281615	*8q24.21*	Intergenic	8	128424800	G/A	g	0.43/0.50	0.107	0.03	1.11 (1.05-1.18)	3.0 × 10^-4^	++++--	0.01
rs999737	*RAD51L1*	Intronic	14	68104435	C/T	c	0.77/0.80	0.079	0.035	1.08 (1.01-1.16)	0.024	++++-+	0.31
rs3817198	*LSP1*	Intronic	11	1865582	C/T	c	0.32/0.34	0.049	0.032	1.05 (0.99-1.12)	0.13	+++-++	0.45
rs1045485	*CASP8*	Missense	2	201857834	G/C	g	0.88/0.92	0.067	0.045	1.07 (0.98-1.17)	0.13	++--++	0.75

^a^Nearest gene(s); ^b^the risk allele refers to the allele associated with a younger age at menarche, an older age at natural menopause, or an increased risk of breast cancer; ^c^effect allele frequency; ^d^effect allele frequency in case or control subjects, respectively; ^e^per effect allele change in log odds ratio (OR) of breast cancer; ^f^*P *value from meta-analysis with additive genetic coding after adjustment for age and top genetic principle components; ^g^direction of effect allele association with breast cancer in the six studies in order: Nurses' Health Study, Women's Genome Health Study, SardiNIA Breast Cancer Study, Rotterdam Study I and II, Framingham Heart Study, and Atherosclerosis Risk in Communities Study; ^h^*P *value from heterogeneity test across studies with 5 degrees of freedom. CI, confidence interval; SE, standard error; SNP, single-nucleotide polymorphism.

To evaluate the combined effect of candidate SNPs on breast cancer risk, we calculated a GRS for each trait by using either a count GRS or a weighted GRS approach. The mean values of count and weighted GRSs were 20.41 and 20.03, respectively, for age at menarche and 16.21 and 14.32, respectively, for age at natural menopause (Table 3). Based on the count GRS for a younger age at menarche, the OR for breast cancer associated with each point scored, corresponding to 1 risk allele, was 1.01 (95% CI = 1.00 to 1.03) after age and potential population stratification were adjusted for. ORs did not increase linearly across quintiles of GRS for age at menarche (*P *for trend = 0.06). Compared with women in the lowest quintile, women in the fourth and fifth quintiles had ORs for breast cancer of 1.14 (95% CI = 1.01 to 1.28) and 1.13 (95% CI = 1.00 to 1.27), respectively. Results were similar when analyses were performed by using weighted GRS. Overall, we did not observe statistically significant associations between breast cancer risk and age at natural menopause when either count or weighted GRS was used.

**Table 3:** Association between genetic risk score and risk of breast cancer

		Quintile of GRS					
	Continuous GRS	Quintile 1	Quintile 2	Quintile 3	Quintile 4	Quintile 5	*P *for trend
Count GRS^a^							
Age at menarche							
Cases/Controls	3,683/34,174	677/6,694	739/6,814	731/6,788	783/6,915	753/6,963	
Mean/Median GRS (range)^b^	20.41 (9.12-31.95)	17.00 (9.12-18.09)	19.02 (18.09-19.86)	20.37 (19.86-21.05)	21.90 (21.05-22.74)	23.89 (22.74-31.95)	
OR (95% CI)^c^	1.01 (1.00-1.03)	1.00	1.10 (0.97-1.24)	1.01 (0.89-1.14)	1.14 (1.01-1.28)	1.13 (1.00-1.27)	0.06
Age at natural menopause							
Cases/Controls	3,683/34,174	740/6,699	751/6,948	719/6,787	755/6,929	718/6,811	
Mean/Median GRS (range)^b^	16.21 (4.15-26.52)	12.95 (4.15-14.01)	14.91 (14.01-15.62)	16.15 (15.62-16.90)	17.64 (16.90-18.37)	19.55 (18.37-26.52)	
OR (95% CI)^c^	1.00 (0.98-1.01)	1.00	1.02 (0.90-1.15)	0.93 (0.82-1.05)	1.02 (0.90-1.14)	0.98 (0.87-1.11)	0.54
Breast cancer							
Cases/Controls	3,683/34,174	537/6,864	542/6,525	660/6,716	956/7,352	988/6,717	
Mean/Median GRS (range)^b^	8.88 (1.06-16.96)	6.04 (1.06-7.01)	7.99 (7.01-8.05)	8.99 (8.05-9.07)	9.99 (9.07-10.86)	11.31 (10.86-16.96)	
OR (95% CI)^c^	1.13 (1.11-1.15)	1.00	1.19 (1.04-1.37)	1.38 (1.21-1.57)	1.60 (1.41-1.81)	1.89 (1.67-2.14)	1.5 × 10^-31^
Weighted GRS^a^							
Age at menarche							
Cases/Controls	3,683/34,174	762/8,961	711/6,811	752/6,287	728/6,059	736/6,056	
Mean/Median GRS (range)^b^	20.03 (7.15-32.17)	16.12(7.15-17.50)	18.47 (17.50-19.31)	20.07 (19.31-20.83)	21.63 (20.83-22.55)	23.82 (22.55-32.17)	
OR (95% CI)^c^	1.01 (1.00-1.03)	1.00	1.02 (0.90-1.15)	0.99 (0.88-1.13)	1.02 (0.90-1.16)	1.11 (0.98-1.26)	0.12
Age at natural menopause							
Cases/Controls	3,683/34,174	749/7,038	728/6,915	756/6,749	696/6,668	755/6,804	
Mean/Median GRS (range)^b^	14.32 (2.78-26.21)	11.13 (2.78-12.18)	12.96 (12.18-13.61)	14.24 (13.61-14.88)	15.57 (14.88-16.39)	17.63 (16.39-26.21)	
OR (95% CI)^c^	1.00 (0.98-1.01)	1.00	1.07 (0.95-1.20)	0.97 (0.86-1.09)	1.00 (0.88-1.13)	1.03 (0.91-1.16)	0.73

^a^See the 'Materials and methods' section for count genetic risk score (GRS) and weighted GRS computation; ^b^mean for continuous GRS and median for each quintile; ^c^adjusted for age and potential population stratification. CI, confidence interval; OR, odds ratio.

**Figure 2:**
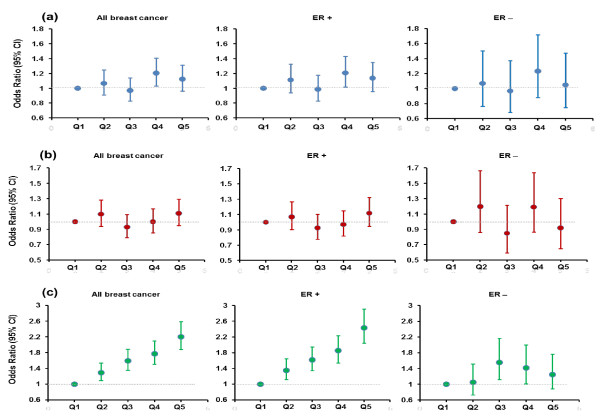
**The associations between groups defined by quintiles of genetic risk scores (GRSs) and risk of breast cancer by estrogen receptor (ER) status in the Nurses' Health Study and the Women's Genome Health Study**. **(a) **Count GRS for age at menarche. **(b) **Count GRS for age at natural menopause. **(c) **Count GRS for breast cancer-associated SNPs. CI, confidence interval.

In secondary analyses, we then determined whether the associations of the 46 candidate SNPs with breast cancer vary across tumor subtypes defined by ER status in the NHS and the WGHS (Table 4). For the two SNPs (rs1079866 and rs7821178) that had reported associations with age at menarche and that were associated with overall breast cancer risk, we found no statistically significant evidence that the associations differed across subtypes (*P *for heterogeneity = 0.31 and 0.66, respectively), although rs1079866 appeared to have a stronger association with ER^+ ^tumors (per-allele OR = 1.26; 95% CI = 1.12 to 1.41) than with ER^- ^tumors (per-allele OR = 1.11; 95% CI = 0.89 to 1.38). Of note, one SNP that had a reported association with age at menarche, rs17188434, had a significantly stronger association with ER^- ^tumors (per-allele OR = 1.51; 95% CI = 1.15 to 1.98) than with ER^+ ^tumors (per-allele OR = 1.08; 95% CI = 0.92 to 1.26; *P *for heterogeneity = 0.035). Another SNP that had a reported association with age at menarche, rs17268785, was associated with a decreased risk of ER^- ^tumors (per-allele OR = 0.83; 95% CI = 0.68 to 1.00) but an increased risk of ER^+ ^tumors (per-allele OR = 1.07; 95% CI = 0.96 to 1.19; *P *for heterogeneity = 0.023). For the SNP that had a reported association with age at natural menopause and that was associated with overall breast cancer risk, we observed a stronger association with ER^- ^tumors (per-allele OR = 1.32; 95% CI = 1.09 to 1.61) than ER^+ ^tumors (per-allele OR = 1.11; 95% CI = 1.00 to 1.24); however, the test for heterogeneity was not statistically significant (*P *for heterogeneity = 0.12). When the count GRS for age at menarche or age at natural menopause was applied to ER^+ ^and ER^- ^breast cancer separately, the trend in the OR for ER^+ ^tumors was very similar to that for overall breast cancer. The ER^- ^tumor data suggested a somewhat different pattern, although the statistical power was limited for this subtype (Figure 2).

**Table 4:** Association of candidate single-nucleotide polymorphism loci and risk of breast cancer by estrogen receptor status in the Nurses' Health Study and Women's Genome Health Study

SNP	All cases		ER^+^		ER^-^		ER^+^/ER^-^
	OR (95% CI)^a^	*P *value^b^	OR (95% CI)^a^	*P *value^b^	OR (95% CI)^a^	*P *value^b^	*P *value for heterogeneity^c^
Cases/Controls^d^	2,087/23,319		1,716/23,319		371/23,319		
Age at menarche							
rs1079866	1.22 (1.10-1.35)	2.0 × 10^-4^	1.26 (1.12-1.41)	7.3 × 10^-5^	1.11 (0.89-1.38)	0.34	0.31
rs7821178	1.12 (1.04-1.20)	3.3 × 10^-3^	1.10 (1.01-1.19)	0.025	1.14 (0.98-1.34)	0.10	0.66
rs17188434	1.15 (1.00-1.33)	0.048	1.08 (0.92-1.26)	0.34	1.51 (1.15-1.98)	2.7 × 10^-3^	0.035
rs13187289	0.92 (0.84-1.00)	0.055	0.90 (0.82-0.99)	0.026	1.05 (0.87-1.28)	0.60	0.15
rs10980926	0.93 (0.87-1.00)	0.067	0.94 (0.87-1.02)	0.14	0.90 (0.77-1.05)	0.17	0.58
rs7642134	1.06 (0.99-1.14)	0.12	1.07 (0.99-1.16)	0.089	1.09 (0.93-1.27)	0.28	0.86
rs10423674	1.05 (0.97-1.13)	0.22	1.06 (0.97-1.15)	0.19	0.95 (0.81-1.11)	0.50	0.23
rs6589964	0.95 (0.89-1.03)	0.22	0.94 (0.87-1.02)	0.13	1.00 (0.85-1.17)	0.98	0.51
rs1398217	1.04 (0.97-1.12)	0.25	1.02 (0.95-1.10)	0.57	1.11 (0.95-1.29)	0.19	0.36
rs1659127	1.03 (0.96-1.12)	0.41	1.00 (0.92-1.09)	0.98	1.09 (0.92-1.29)	0.32	0.38
rs12617311	0.97 (0.89-1.05)	0.44	1.00 (0.91-1.09)	0.93	0.94 (0.79-1.12)	0.48	0.56
rs17268785	1.04 (0.94-1.14)	0.48	1.07 (0.96-1.19)	0.22	0.83 (0.68-1.00)	0.055	0.023
rs2090409	0.97 (0.90-1.05)	0.50	0.96 (0.89-1.05)	0.38	0.98 (0.84-1.15)	0.82	0.85
rs10899489	1.03 (0.93-1.14)	0.52	1.02 (0.92-1.14)	0.67	0.98 (0.79-1.21)	0.86	0.72
rs6438424	0.98 (0.91-1.05)	0.55	0.99 (0.92-1.07)	0.79	0.98 (0.84-1.14)	0.76	0.88
rs2002675	1.02 (0.95-1.10)	0.55	1.04 (0.96-1.12)	0.35	0.94 (0.81-1.10)	0.45	0.27
rs466639	1.02 (0.92-1.14)	0.73	1.05 (0.94-1.18)	0.39	0.94 (0.74-1.20)	0.62	0.41
rs7759938	1.01 (0.93-1.09)	0.84	1.02 (0.94-1.11)	0.63	1.00 (0.84-1.17)	0.96	0.80
rs9635759	1.01 (0.93-1.09)	0.88	1.00 (0.92-1.09)	0.98	1.03 (0.87-1.22)	0.72	0.74
Age at natural menopause							
rs2517388	1.14 (1.03-1.25)	0.010	1.11 (1.00-1.24)	0.046	1.32 (1.09-1.61)	4.7 × 10^-3^	0.12
rs2303369	0.93 (0.86-1.00)	0.036	0.93 (0.86-1.01)	0.076	0.94 (0.80-1.09)	0.42	0.94
rs12461110	0.93 (0.86-1.00)	0.042	0.91 (0.84-0.99)	0.022	0.95 (0.81-1.11)	0.50	0.66
rs4886238	1.06 (0.99-1.14)	0.11	1.06 (0.98-1.15)	0.14	1.03 (0.88-1.21)	0.68	0.76
rs16991615	1.09 (0.94-1.26)	0.23	1.13 (0.97-1.32)	0.12	0.99 (0.71-1.37)	0.94	0.46
rs2277339	1.07 (0.95-1.20)	0.28	1.09 (0.96-1.24)	0.21	1.02 (0.80-1.31)	0.85	0.68
rs12294104	1.04 (0.95-1.14)	0.41	1.06 (0.96-1.17)	0.28	0.98 (0.80-1.21)	0.89	0.54
rs4246511	1.03 (0.95-1.12)	0.42	1.05 (0.96-1.15)	0.29	0.95 (0.79-1.14)	0.59	0.34
rs10852344	1.03 (0.96-1.10)	0.42	1.04 (0.96-1.12)	0.31	0.99 (0.85-1.15)	0.88	0.56
rs2153157	1.03 (0.96-1.10)	0.45	1.04 (0.96-1.12)	0.32	1.00 (0.87-1.17)	0.95	0.69
rs4693089	0.98 (0.91-1.05)	0.54	0.98 (0.91-1.07)	0.68	0.91 (0.77-1.06)	0.23	0.37
rs10183486	0.98 (0.91-1.06)	0.67	0.98 (0.91-1.06)	0.64	0.91 (0.78-1.06)	0.23	0.39
rs2307449	0.99 (0.92-1.06)	0.72	0.96 (0.89-1.04)	0.36	1.06 (0.91-1.24)	0.47	0.29
rs1635501	0.99 (0.91-1.07)	0.76	0.99 (0.90-1.08)	0.80	0.99 (0.83-1.19)	0.94	0.97
rs11668344	1.01 (0.94-1.08)	0.81	1.00 (0.92-1.08)	0.92	1.05 (0.89-1.22)	0.58	0.59
rs1046089	0.99 (0.92-1.07)	0.89	1.02 (0.94-1.10)	0.67	0.90 (0.77-1.05)	0.19	0.17
rs365132	1.00 (0.93-1.07)	0.97	1.00 (0.93-1.08)	1.00	1.00 (0.86-1.16)	0.95	0.96
Breast cancer							
rs11249433	1.20 (1.12-1.29)	4.8 × 10^-7^	1.23 (1.14-1.33)	6.9 × 10^-8^	1.06 (0.91-1.23)	0.45	0.081
rs3803662	1.20 (1.12-1.30)	1.8 × 10^-6^	1.26 (1.16-1.37)	4.2 × 10^-8^	0.98 (0.82-1.16)	0.78	0.008
rs2981582	1.18 (1.10-1.27)	8.2 × 10^-6^	1.19 (1.10-1.29)	1.1 × 10^-5^	1.01 (0.87-1.19)	0.86	0.071
rs13281615	1.16 (1.08-1.24)	3.9 × 10^-5^	1.16 (1.07-1.25)	2.1 × 10^-4^	1.15 (0.99-1.33)	0.074	0.94
rs13387042	1.15 (1.07-1.23)	8.4 × 10^-5^	1.17 (1.08-1.26)	6.3 × 10^-5^	1.01 (0.87-1.18)	0.85	0.10
rs7716600	1.17 (1.08-1.27)	1.6 × 10^-4^	1.18 (1.08-1.29)	2.9 × 10^-4^	1.21 (1.02-1.44)	0.029	0.77
rs889312	1.10 (1.02-1.19)	0.012	1.10 (1.01-1.20)	0.026	1.18 (1.00-1.39)	0.046	0.45
rs999737	1.09 (1.00-1.18)	0.049	1.10 (1.01-1.21)	0.031	0.99 (0.84-1.18)	0.95	0.29
rs1045485	1.09 (0.98-1.20)	0.12	1.06 (0.95-1.19)	0.28	1.20 (0.95-1.52)	0.12	0.35
rs3817198	1.03 (0.96-1.12)	0.38	1.06 (0.98-1.15)	0.16	0.88 (0.74-1.04)	0.13	0.047

^a^Per-risk-allele odds ratio (OR) of breast cancer; ^b^*P *value from meta-analysis of the Nurses' Health Study (NHS) and the Women's Genome Health Study (WGHS) with additive genetic coding after adjustment for age and potential population stratification; ^c^*P *value from heterogeneity test across estrogen receptor-positive (ER^+^) and ER^- ^tumors; ^d^total number of cases or controls in the NHS and the WGHS. CI, confidence interval; SNP, single-nucleotide polymorphism.

Of the 10 candidate SNPs with consistently reported associations with breast cancer risk, five SNPs (rs11249433, rs3803662, rs2981582, rs13387042, and rs999737) appeared to have a stronger association with ER^+ ^tumor than ER^- ^tumors, and rs3803662 reached statistical significance (*P *for heterogeneity = 0.008) with per-risk-allele ORs of 1.26 (95% CI = 1.16 to 1.37) and 0.98 (95% CI = 0.82 to 1.16) for ER^+ ^and ER^- ^tumors, respectively (Table 4). Two breast cancer candidate SNPs, rs1045485 and rs3817198, did not show statistically significant associations with overall risk. However, rs1045485 appeared to have a stronger association with ER^- ^tumors (per-allele OR = 1.20; 95% CI = 0.95 to 1.52) than ER^+ ^tumors (per-allele OR = 1.06; 95% CI = 0.95 to 1.19; *P *for heterogeneity = 0.35), and rs3817198 was associated with a decreased risk of ER^- ^tumors (per-allele OR = 0.88; 95% CI = 0.74 to 1.04) but an increased risk of ER^+ ^tumors (per-allele OR = 1.06; 95% CI = 0.98 to 1.15; *P *for heterogeneity = 0.047).

In these analyses, we further confirmed statistically significant associations with breast cancer risk for 8 of the 10 candidate SNPs that were identified previously in published GWAS of breast cancer (most *P *values were less than 0.001) (Table 2). We did not observe a statistically significant association for either *LSP1*-rs3817198 or *CASP8*-rs1045485 (both with *P *values of 0.13) in our study, although the direction of the associations was consistent with that of previous reports [21,22]. We also calculated, as a positive control, a count GRS based on these 10 SNPs. We found that each score point increase, corresponding to one-risk-allele increase, was significantly associated with an OR of 1.13 (95% CI = 1.11 to 1.15) for breast cancer (Table 3). Compared with women in the lowest quintile, women in the highest quintile had an OR for breast cancer of 1.89 (95% CI = 1.67 to 2.14). For this GRS, the trend in log odds was significantly steeper for ER^+ ^than for ER^- ^tumors (*P *for heterogeneity < 0.001), and the OR across quintiles was no longer monotonic in ER^- ^tumors (Figure 2).

## Discussion

In this large meta-analysis of six population-based studies, we investigated whether 19 loci linked with age at menarche and 17 loci linked with age at natural menopause were associated with breast cancer risk among up to 3,683 breast cancer cases and 34,174 controls. We found that two SNPs with reported associations with age at menarche and one SNP with a reported association with age at natural menopause were significantly associated with breast cancer risk. However, no statistically significant associations were found for GRSs that combined all 19 or 17 loci associated with each trait, although the association for age-at-menarche GRS was marginally statistically significant. We confirmed most of the candidate loci for breast cancer which were identified in previous GWAS. Some of these associations appeared to differ by tumor subtypes defined by ER status.

In our analyses, most of the candidate SNPs, including the strongest GWAS hit for age at menarche or age at natural menopause, were not found to be associated with breast cancer risk. This is not necessarily surprising given that age at menarche and age at natural menopause are relatively weak risk factors [2,3], and all candidate SNPs collectively explain only a small portion of the variation of each trait [10,11]. However, two candidate SNPs for age at menarche, rs1079866 and rs7821178, and one candidate SNP for age at natural menopause, rs2517388, were found to be associated with breast cancer risk. These associations were not attenuated after we further adjusted for self-reported age at menarche and age at natural menopause, suggesting these three genetic loci were associated with breast cancer risk independently of their associations with age at menarche or age at natural menopause. It is possible that these genetic loci have pleiotropic effects on reproductive timing as well as other biological processes leading to breast cancer, and the observed associations might be due largely to other biological consequences of these risk variants that do not manifest themselves as changes in age at menarche or age at natural menopause. Alternatively, it is also possible that the relatively crude assignment of these reproductive events to a single chronological year is not sufficiently accurate to capture the biological effect of these processes on breast cancer risk and the genetic variants contribute independent information on the underlying biological risk. The three candidate SNPs also contributed to breast cancer risk independently of the known susceptibility loci for breast cancer, as further adjustment for breast cancer loci did not materially alter the results.

We found statistically significant evidence of association with breast cancer for eight of the 10 breast cancer susceptibility loci examined: *FGFR2*-rs2981582, *TNRC9*-rs3803662, 1p-rs11249433, 5p-rs7716600, 2q35-rs13387042, *MAP3K1*-rs889312, 8q24-rs13281615, and *RAD51L1*- rs999737. The direction and magnitude of these associations were consistent with those of previous reports [17,18,22-25]. We did not observe a statistically significant association for either *LSP1*-rs3817198 or *CASP8*-rs1045485. However, these two SNPs had relatively small reported effects that our study might not have been able to detect. When the 10 candidate SNPs were combined by using a polygenic risk score, the relative risk for women in the highest quintile was about twice that in the lowest quintile, and this is in accordance with other published results [19,26]. In this study, none of the 10 breast cancer susceptibility loci was significantly associated with age at menarche or age at natural menopause, and this is in line with a previous report [27].

Given that most of the candidate loci for age at menarche and age at natural menopause were not associated with breast cancer risk, it is not surprising that there were no statistically significant associations for the polygenetic risk scores that combined all candidate loci for each trait. To conduct a *post hoc *and exploratory analysis, we created a polygenetic risk score by including only the three candidate loci associated with either age at menarche or age at natural menopause and with breast cancer risk and found that each risk allele increment was associated with an approximately 17% increased risk for breast cancer. Women with four or more risk alleles had an approximately 60% increased risk for breast cancer in comparison with those with two risk alleles or less. When we further combined the three associated SNPs with the 10 breast cancer susceptibility loci to create a polygenetic risk score, each risk allele increment was associated with an approximately 18% increased risk for breast cancer. For women with 14 or more risk alleles (the highest quintile), the risk for breast cancer increased threefold in comparison with those with 10 or less (the lowest quintile). Because the former group constitutes approximately 20% of the study population, the GRS that combines the three candidate SNPs for age at menarche and age at natural menopause and the identified breast cancer susceptibility loci might be useful for identifying a subgroup of women with a high genetic risk for breast cancer. Further research is needed to confirm this finding.

It has been hypothesized that the risk of ER^+ ^breast cancer is positively associated with a woman's cumulative lifetime exposure to endogenous ovarian hormones [28]. A younger age at menarche [12,15,29] and an older age at menopause [30] have been observed to be more consistently associated with ER^+ ^than ER^- ^tumors. In this report, we found that candidate loci for age at menarche and age at natural menopause may also be differentially associated with tumor subtypes defined by ER status. Of the three candidate loci that were found to be associated with overall breast cancer risk, rs1079866 was more strongly associated with ER^+ ^tumors, rs7821178 was equally associated with both, whereas rs2517388 was more strongly associated with ER^- ^tumors, although differences were not statistically significant. Importantly, two candidate loci for age at menarche, rs17188434 and rs17268785, had significantly different associations with ER^+ ^and ER^- ^tumors. Whereas both SNPs were not significantly associated with overall and ER^+ ^breast cancer, the former showed a statistically significant positive association with ER^- ^tumors, whereas the latter showed a statistically significant inverse association with ER^- ^tumor. These findings provide further support for the notion that ER^+ ^and ER^- ^tumors are the result of different etiologic pathways [31].

Although common genetic variants that influence the intermediate phenotypes or risk factors have been hypothesized to subsequently affect disease risk, few studies have assessed the association between these genetic variants and disease risk or, furthermore, whether these associations are mediated through the intermediate phenotypes. Chen and colleagues [32] investigated obesity-linked genetic variants in relation to breast cancer risk but found no statistically significant association. To our knowledge, ours is the first study to evaluate the associations of candidate loci for age at menarche and age at natural menopause with breast cancer risk. One of the strengths of our study is the relatively large combined sample size achieved through international collaboration. We had adequate statistical power (80%) to detect an OR of 1.12 for SNPs with a minor allele frequency (MAF) of 0.10 and an OR of 1.09 for SNPs with an MAF of 0.20. However, our analysis of ER^+ ^tumors was less adequately powered, as the ER status was not available for all cases, and the study had limited statistical power for ER^- ^tumors. One limitation in our study is the multiple comparisons that could lead to false-positive results. Although none of the candidate SNPs with a reported association with age at menarche or age at natural menopause survived Bonferroni correction in the test of breast cancer association, this correction is considered to be overly conservative given that the candidates were chosen on the basis of promising hypotheses. Another potential limitation of our study comes from differences in the study population and designs and methods of collecting risk factors and genetic marker data across studies. However, the findings were generally consistent across studies, arguing for the robustness of our results. Finally, as our analyses were restricted to women of European ancestry, results from this study may not be generalizable to other ethnic groups.

## Conclusions

In summary, in this large analysis of the association of several novel candidate loci for age at menarche and age at natural menopause with breast cancer risk, we observed that three loci - two for age at menarche and one for age at natural menopause - were significantly associated with breast cancer risk independently of their associations with each trait and independently of known breast cancer susceptibility loci. These associations may differ by tumor subtypes defined by ER status. A combination of all 19 loci associated with age at menarche or 17 loci associated with age at natural menopause did not appear to be helpful for identifying a high-risk subgroup for breast cancer.

## Abbreviations

ARIC: Atherosclerosis Risk in Communities Study; BMI: body mass index; CI: confidence interval; ER: estrogen receptor; FHS: Framingham Heart Study; GRS: genetic risk score; GWAS: genome-wide association studies; MAF: minor allele frequency; NHS: Nurses' Health Study; OC: oral contraceptive; OR: odds ratio; RSI+II: Rotterdam Study I and II; SardiNIA: SardiNIA Breast Cancer Study; SNP: single-nucleotide polymorphism; WGHS: Women's Genome Health Study.

## Competing interests

The authors declare that they have no competing interests.

## Authors' contributions

CH performed the meta-analysis, wrote the manuscript, helped to conceive and design the experiments, helped to perform the primary statistical analyses in each study, shared responsibility for the interpretation of results and critical revision of the manuscript, and contributed to a critical revision of the manuscript for important intellectual content. DIC helped to conceive and design the experiments, helped to perform the primary statistical analyses in each study, shared responsibility for the interpretation of results and critical revision of the manuscript, and contributed to a critical revision of the manuscript for important intellectual content. DJH helped to conceive and design the experiments, contributed to a critical revision of the manuscript for important intellectual content, and participated in the original design, subject recruitment, acquisition of data, biospecimen collection for the studies, and the genotyping and quality control of genotype and other data. JD, SJH, RR, and SS helped to perform the primary statistical analyses in each study and shared responsibility for the interpretation of results and critical revision of the manuscript. SJC, LC, EWD, JMM, PMR, and BHS contributed to a critical revision of the manuscript for important intellectual content and participated in the original design, subject recruitment, acquisition of data, biospecimen collection for the studies, and the genotyping and quality control of genotype and other data. JEB, LF-R, NF, SEH, AH, KLL, GP, EP, FR, LMR, GLS, LS, and AGU participated in the original design, subject recruitment, acquisition of data, biospecimen collection for the studies, and the genotyping and quality control of genotype and other data. All authors read and approved the final manuscript.

## Supplementary Material

Additional file 1 **Supplementary methods for study population**. Click here for file

Additional file 2 **Table S1: Information on the 46 candidate SNP loci identified in previous genome-wide association studies for age at menarche, age at natural menopause and breast cancer**. Click here for file

Additional file 3 **Table S2: Characteristics of non-genetic risk factors for breast cancer in each participating study**. Click here for file
