# Supplementary material for: Reproductive aging-associated common genetic variants and the risk of breast cancer
Source: Breast Cancer Res. 2012 Mar 20;14(2):R54. doi: 10.1186/bcr3155 (PMC3446388; doi:10.1186/bcr3155)
Supplement: Additional file 1 — Supplementary methods for study population. [file bcr3155-S1.DOCX]

**Supplementary Methods**

**Study Populations**

**NHS:** Details of the Nurses’ Health Study (NHS) cohorts have been described previously [[1](#_ENREF_1)]. Briefly, the NHS was initiated in 1976, when 121,700 United States registered nurses between the ages of 30 and 55, residing in 11 larger U.S. states, returned an initial questionnaire reporting medical histories and baseline health-related exposures, including information related to reproductive history (age at menarche, age at first birth, parity, age at menopause etc.), and exposure to exogenous hormones (oral contraception or post-menopausal hormone replacement therapy). Biennial questionnaires containing exposure information on risk factors have been collected prospectively, and outcome data with follow-up of reported disease events are collected. From May 1989 through September 1990, blood samples were collected from 32,826 participants in the NHS cohort. Subsequent follow-up has been greater than 99% for this subcohort. Informed consent was obtained from all participants. The study was approved by the Institutional Review Board of the Brigham and Women’s Hospital, Boston, MA, USA.

The NHS nested breast cancer case-control study was derived from the 32,826 women in the blood subcohort who were free of diagnosed breast cancer at the time of blood collection, and who were followed for incidence of disease until June 1, 2004. Breast cancer follow-up was conducted by personal mailings and searches of the National Death Index. Controls were women not diagnosed with breast cancer during follow-up. The controls were one-to-one matched to cases based on age at the time of diagnosis, blood collection variables (time of day, season, and year of blood collection, as well as recent (<3 months) use of postmenopausal hormones), ethnicity (all cases and controls are self-reported white women of European ancestry), and menopausal status (all cases were postmenopausal at diagnosis). The 2,287 NHS participants included in the present analysis were from this nested breast cancer case-control study and were self-described white women of European ancestry with genotype data available from the National Cancer Institute’s Cancer Genetic Marker of Susceptibility (CGEMS) project [[2](#_ENREF_2)]. The GWAS scan used the Illumina Infinium Sentrix HumanHap550 chip. Detailed methods related to the genotyping have been published previously [[2](#_ENREF_2)]. Imputation of untyped genotypes of about 2.5 million SNP was based on HapMap haplotype reference (HapMap Phase II CEU population, Release 21, NCBI build 35) using MACH software[[3](#_ENREF_3)].

**WGHS:** The Women’s Genome Health Study (WGHS) is a prospective cohort of female healthcare professionals, aged 45 or older at baseline, who provided baseline blood samples and consent for blood based analysis in the Women’s Health Study (WHS), a randomized, placebo controlled trial of aspirin and vitamin E for the primary prevention of cardiovascular disease and cancer. A complete description of the WGHS has been published previously [[4](#_ENREF_4)]. The study was approved by the Institutional Review Board of the Brigham and Women’s Hospital, Boston, MA, USA. All information about reproductive aging was self-reported in a questionnaire at baseline and at subsequent follow-ups. Additional information related to health and lifestyle were collected by questionnaire throughout the WHS trial and continuing observational follow-ups. Every 6 months during the first year of follow-up and then annually thereafter, participants were asked whether they had been newly diagnosed with breast cancer. We then confirmed the diagnosis for women who reported a diagnosis of breast cancer and those who were deceased through a review of medical records and extracted detailed information on the diagnosis of breast cancer. In the present study, we included 1099 women who had a confirmed diagnosis of invasive breast cancer during an average follow-up of 17 years between 1992 and 2009.

Genotyping in the WGHS sample was performed using the HumanHap300 Duo ‘‘+’’ chips or the combination of the HumanHuman300 Duo and iSelect chips (Illumina, San Diego, CA) using the Infinium II protocol. In either case, the custom SNP content was the same; these custom SNPs were chosen without regard to minor allele frequency(MAF) to saturate candidate genes for cardiovascular disease as well as to increase coverage of SNPs with known or suspected biological function, e.g. disease association, non-synonymous changes, substitutions at splice sites, etc. For quality control, all samples were required to have successful genotyping using the Bead Studio v.3.3 software (Illumina, San Diego, CA) for at least 98% of the SNPs. A subset of 23,294 individuals were identified with self-reported European ancestry that could be verified on the basis of multidimensional scaling analysis of identity by state using1443 ancestry informative markers in PLINK v. 1.06. In the final dataset of these individuals, SNPs were retained with MAF >1%, successful genotyping in 90% of the subjects, and deviations from the Hardy-Weinberg equilibrium not exceeding P=10^-6^ in significance. Among the final 23,294 individuals of verified European ancestry, genotypes for a total of 2,608,509 SNPs were imputed from the experimental genotypes and LD relationships implicit in the HapMap release 22 CEU samples.

**SardBC:** The SardBC study recruited a total of 1,431 unselected Sardinian breast cancer patients from University clinics and Hospitals across the Sardinia Island (University of Cagliari and Sassari, ASL1 of Sassari and Oncologic Hospital of Cagliari). Sardinian origin was ascertained in all cases through genealogical information (at least 3 of the four grandparents were born in Sardinia). Family history of cancer was evaluated through specific questionnaires during follow-up visits at the participating institutions. Some of the patients had a family history of breast/ovarian cancer, thus fulfilling the selection criterion for "high risk" families. “High-risk” families are defined as those where one member was diagnosed with breast cancer before the age of 50.In addition, these individuals have at least one first or second-degree relative also diagnosed with breast cancer before the age 50 or ovarian cancer at any age. The remaining cases were categorized as cases from "no high risk" families: presence of a breast cancer patient having at least one first or second-degree relative who is diagnosed with breast cancer or ovarian cancer at any age, and as sporadic cases, with no familiarity.

All high risk patients included in the study were found to be negative for the *BRCA1* and *BRCA2* mutations. Clinical and pathological characteristics of the breast cancer probands including age at diagnosis, mono- or bilateral tumor location, stage of the disease, tumor-node metastasis system status, and tumor grading, were confirmed by medical records and the pathology reports for a subset of the patients. A written informed consent was obtained for both genetic counseling and blood/tissue sampling before the genetic test was conducted. The study was reviewed and approved by the ethical review boards.

Controls(N=2,171) were recruited in part from the blood donor centers for a parallel case-control study on autoimmune diseases [[5](#_ENREF_5)]; in part from a family-based study on asthma(parents of asthma probands for whom absence of breast cancer was self-reported)[[6](#_ENREF_6)], and in part from the blood donors matched for age and ethnicity and with no diagnosis of cancer or any other chronic diseases attending the Transfusional Services at both the University and ASL1 of Sassari. As for the cases, Sardinian origin was ascertained based on family history.

This study was conducted with different numbers of volunteers and genotyping platforms. In particular, we genotyped 740 cases and 412 controls with the Affymetrix 500K array, and 688 cases and 921 controls with the Affymetrix 6.0 array. Samples were discarded if the overall call rate was <90%, and if the reported gender was differed from the genotype data of the X and Y chromosomes. We then used IBD sharing to detect, and subsequently discard, hidden relatives using RELPAIR[[7](#_ENREF_7)], and principal component analysis to remove outliers using the Smartpca in Eigensoft package[[8](#_ENREF_8)].The final sample size available for analysis consisted of 1,387 cases and 1,141 controls. At the SNP level, we applied the following quality filters: Call rate < 95%, MAF <5%, HWE p-value <10-6, and, for markers included on both platforms (~500,000) we required that frequency estimates were similar (MAF <0.1). We then considered only common autosomal SNPs between the two platforms(328,705) and performed imputation with MACH[[3](#_ENREF_3)] software using HapMap2 CEU build 36 haplotypes. Association analysis was then performed adjusting for the principal component axes.

**RSI+II:** Rotterdam Study I and II **(**RSI+II) are ongoing prospective population-based cohort studies of whites of European ancestry aged 55 years and older, living in the Ommoord district of Rotterdam, the Netherlands [[9](#_ENREF_9)]. The study was designed to investigate the incidence and determinants of chronic disabling diseases in the elderly. The rationale and design have been described previously [[9](#_ENREF_9)]. For RSI, all 10,275 aged 55 years and over were invited for baseline examination between August 1990 and June 1993, of those, 7,983 participated. In 1999, 3,011 participants (out of 4,472 invitees) were added to the cohort (RSII). These participants had either reached the age of 55 or moved into the study district since the start of the study. Questionnaires including menarche, menopause and parity related questions as well as questions concerning alcohol consumption, postmenopausal hormone use, oral contraceptive use, and the number of relatives with breast cancer were filled out by study nurses during the home interview. Blood samples were taken from over 70 percent of the participants at the research center. Diagnoses of breast cancer were obtained from general practitioners and by linkage with a nationwide registry of histo- and cytopathology in the Netherlands (PALGA) from January 1^st^, 1986 onwards. Two research physicians independently assessed the first date and diagnosis of breast cancer. All events were classified according to the International Classification of Disease(ICD) [[10](#_ENREF_10)]. In case of discrepancies of diagnosis, decisions were reached either through consensus or by a cancer epidemiologist. The current study is based on 3,342 women from RSI and 1,135 from RSII for whom GWAS data and age at natural menopause or age at menarche were available. Of these, 181 and 35 participants, respectively, were diagnosed with breast cancer.The Rotterdam Study was approved by the medical ethics committee of the Erasmus University Medical School, and written informed consent was obtained from each subject. Single SNP analyses as well as genetic risk score analyses were performed using SPSS software (version 15.0, Chicago, US).

**FHS:** The Framingham Heart Study (FHS) is a multi-generational community-based longitudinal cohort study [[11](#_ENREF_11)] . The original cohort includes 2873 women (mean age 44 years at enrollment) who were examined every two years since recruitment in 1948. The Offspring cohort, the children of the original cohort members and spouses of the children, includes 2641 women (mean age 36 years at enrollment) who were examined every 4 to 8 years since the study began in 1971. From 2002 to 2005 the Third Generation cohort was enrolled which includes 2182 women with a mean age 40 years. Self-reported cases of cancer were identified at the time of routine research examinations or by health history updates for participants who did not attend an exam. The majority of cancers were confirmed by pathology reports (<5% of cases were based on death certificate or clinical diagnosis). The date of diagnosis and location, histology and morphology, and behavior were recorded. The 1976 World Health Organization ICD-O coding was used to classify all primary cancers. Breast cancer cases reviewed through December 2008 were included in this study. Women with a diagnosis of breast cancer prior to study entry (n=11) were excluded. Among the 4586 Framingham women with GWAS data, we identified 207 women with a diagnosis of breast cancer (cases) and 3698 women who were cancer-free and neither had a mother or sister in the study with breast cancer (controls). Logistic regression using GEE to account for family relation was used to test for association between each SNP or genetic risk score and breast cancer (outcome) using an additive genetic model. Covariates in the model included age, Offspring cohort (yes/no), Third Generation cohort (yes/no) and principal components. The Institutional Review Board at Boston University Medical Center approved the examination content of the Framingham Heart Study Original Cohort, Offspring Cohort and Third Generation Cohort examinations. All participants provided written informed consent including consent for genetic studies.

**ARIC:** The Atherosclerosis Risk in Communities(ARIC) study is a multi-center prospective investigation of atherosclerotic disease in a bi-racial population [[12](#_ENREF_12)]. Men and women aged 45-64 years at baseline were recruited from 4 communities: Forsyth County, North Carolina; Jackson, Mississippi; suburban areas of Minneapolis, Minnesota; and Washington County, Maryland. A total of 15,792 individuals participated in the baseline examination from 1987-1989, with four follow-up examinations in approximate 3-year intervals, during 1990-1992, 1993-1995, and 1996-1998. Only white women of European ancestry with genotype data and age at menarche between 9 and 17 years were included in this analysis (N=4247). This study was approved by the institutional review board at each field center. The analysis was approved by the School of Public Health Institutional Review Board of the University of North Carolina at Chapel Hill School for research involving human subjects. All subjects provided written informed consent.

The ARIC sample we used for this analysis included N= 207 breast cancer cases and N= 2194 controls for which GWA data were available. The age range was from 44to 65 years. For our study we have included only white women of European ancestry women with an age at menarche between 9-17years inclusive, and age at natural menopause between 40-60 years inclusive. Controls for our study were women who were free of breast cancer and who met the above inclusion criteria. Incident breast cancer cases were ascertained from 1987-2000 using linkages to the following cancer registries: Maryland Cancer Registry, Minnesota Cancer Surveillance System, Mississippi Central Cancer Registry (from 1995), North Carolina Cancer Registry, and the Washington County (Maryland) Cancer Registry. Review of all ICD codes of hospitalizations reported by the ARIC study and medical record retrieval was used to identify breast cancer cases that became incident prior to the initiation of cancer registries in Jackson Mississippi(from 1987-1995) and in Minnesota(from 1987-1988), as well as to catch other cases that might have been missed . The covariates of age, age at menarche, age at natural menopause, parity, alcohol consumption (alcohol intake (g) per day at baseline visit), hormone use (self-reported at baseline visit), and oral contraceptive use were all self-reported at the baseline visit. Weight and height were measured at the baseline clinic visit while participants were dressed in scrub suits and had their shoes removed. Body mass index (BMI) was calculated as weight/height^2^ (kg/m^2^).

**References**

1. Colditz GA, Hankinson SE: **The Nurses' Health Study: lifestyle and health among women**. *Nat Rev Cancer* 2005, **5**:388-396.

2. Hunter DJ, Kraft P, Jacobs KB, Cox DG, Yeager M, Hankinson SE, Wacholder S, Wang Z, Welch R, Hutchinson A, Wang J, Yu K, Chatterjee N, Orr N, Willett WC, Colditz GA, Ziegler RG, Berg CD, Buys SS, McCarty CA, Feigelson HS, Calle EE, Thun MJ, Hayes RB, Tucker M, Gerhard DS, Fraumeni JF, Jr., Hoover RN, Thomas G, Chanock SJ: **A genome-wide association study identifies alleles in FGFR2 associated with risk of sporadic postmenopausal breast cancer**. *Nat Genet* 2007, **39**:870-874.

3. Li Y, Willer CJ, Ding J, Scheet P, Abecasis GR: **MaCH: using sequence and genotype data to estimate haplotypes and unobserved genotypes**. *Genet Epidemiol* 2010, **34**:816-834.

4. Ridker PM, Chasman DI, Zee RY, Parker A, Rose L, Cook NR, Buring JE: **Rationale, design, and methodology of the Women's Genome Health Study: a genome-wide association study of more than 25,000 initially healthy american women**. *Clin Chem* 2008, **54**:249-255.

5. Sanna S, Pitzalis M, Zoledziewska M, Zara I, Sidore C, Murru R, Whalen MB, Busonero F, Maschio A, Costa G, Melis MC, Deidda F, Poddie F, Morelli L, Farina G, Li Y, Dei M, Lai S, Mulas A, Cuccuru G, Porcu E, Liang L, Zavattari P, Moi L, Deriu E, Urru MF, Bajorek M, Satta MA, Cocco E, Ferrigno P *et al*: **Variants within the immunoregulatory CBLB gene are associated with multiple sclerosis**. *Nat Genet* 2010, **42**:495-497.

6. Balaci L, Spada MC, Olla N, Sole G, Loddo L, Anedda F, Naitza S, Zuncheddu MA, Maschio A, Altea D, Uda M, Pilia S, Sanna S, Masala M, Crisponi L, Fattori M, Devoto M, Doratiotto S, Rassu S, Mereu S, Giua E, Cadeddu NG, Atzeni R, Pelosi U, Corrias A, Perra R, Torrazza PL, Pirina P, Ginesu F, Marcias S *et al*: **IRAK-M is involved in the pathogenesis of early-onset persistent asthma**. *Am J Hum Genet* 2007, **80**:1103-1114.

7. Epstein MP, Duren WL, Boehnke M: **Improved inference of relationship for pairs of individuals**. *Am J Hum Genet* 2000, **67**:1219-1231.

8. Price AL, Patterson NJ, Plenge RM, Weinblatt ME, Shadick NA, Reich D: **Principal components analysis corrects for stratification in genome-wide association studies**. *Nat Genet* 2006, **38**:904-909.

9. Hofman A, Breteler MM, van Duijn CM, Janssen HL, Krestin GP, Kuipers EJ, Stricker BH, Tiemeier H, Uitterlinden AG, Vingerling JR, Witteman JC: **The Rotterdam Study: 2010 objectives and design update**. *Eur J Epidemiol* 2009, **24**:553-572.

10. WHO: **World Health Organization • International Statistical Classification of Diseases and Related Health Problems, 10th revision** In*.*; 1992.

11. Splansky GL, Corey D, Yang Q, Atwood LD, Cupples LA, Benjamin EJ, D'Agostino RB, Sr., Fox CS, Larson MG, Murabito JM, O'Donnell CJ, Vasan RS, Wolf PA, Levy D: **The Third Generation Cohort of the National Heart, Lung, and Blood Institute's Framingham Heart Study: design, recruitment, and initial examination**. *Am J Epidemiol* 2007, **165**:1328-1335.

12. **The Atherosclerosis Risk in Communities (ARIC) Study: design and objectives. The ARIC investigators**. *Am J Epidemiol* 1989, **129**:687-702.
