# Supplementary material for: Reproductive aging-associated common genetic variants and the risk of breast cancer
Source: Breast Cancer Res. 2012 Mar 20;14(2):R54. doi: 10.1186/bcr3155 (PMC3446388; doi:10.1186/bcr3155)
Supplement: Additional file 2 — Table S1: Information on the 46 candidate SNP loci identified in previous genome-wide association studies for age at menarche, age at natural menopause and breast cancer. [file bcr3155-S2.DOCX]

**Table S1.Information on the 46 candidate SNP loci identified in previous genome-wide association studies for age at menarche, age at natural menopause and breast cancer.**

| **SNP** | **Risk (coded/effect) Allele^a^** | **Frequency of Risk Allele** | **Weight for Risk Allele^b^** | **Ref. (noncoded/Ref.) Allele** | **Imputation Quality^c^** |
| --- | --- | --- | --- | --- | --- |
| **Age at Menarche^d^** | |  |  |  |  |
| rs10423674 | C | 0.65 | 0.0298 | A | 0.958 |
| rs1079866 | C | 0.85 | 0.0324 | G | 0.940 |
| rs10899489 | C | 0.85 | 0.0274 | A | 0.940 |
| rs10980926 | G | 0.64 | 0.0157 | A | 0.997 |
| rs12617311 | A | 0.32 | 0.0485 | G | 0.912 |
| rs13187289 | C | 0.80 | 0.0542 | G | 0.910 |
| rs1398217 | G | 0.43 | 0.0527 | C | 0.979 |
| rs1659127 | G | 0.66 | 0.0426 | A | 0.886 |
| rs17188434 | C | 0.07 | 0.0431 | T | 0.994 |
| rs17268785 | A | 0.83 | 0.0558 | G | 0.984 |
| rs2002675 | A | 0.58 | 0.0472 | G | 0.992 |
| rs2090409 | A | 0.31 | 0.0843 | C | 0.994 |
| rs466639 | T | 0.13 | 0.0558 | C | 0.964 |
| rs6438424 | A | 0.50 | 0.0583 | C | 0.991 |
| rs6589964 | A | 0.48 | 0.0311 | C | 0.890 |
| rs7642134 | A | 0.38 | 0.0546 | G | 0.989 |
| rs7759938 | T | 0.68 | 0.1217 | C | 0.960 |
| rs7821178 | A | 0.34 | 0.0319 | C | 0.983 |
| rs9635759 | G | 0.68 | 0.0496 | A | 0.918 |
|  |  |  |  |  |  |
| **Age at Natural Menopause^d^** | |  |  |  |  |
| rs10183486 | C | 0.63 | 0.1372 | T | 0.983 |
| rs1046089 | G | 0.62 | 0.1811 | A | 1.000 |
| rs10852344 | C | 0.40 | 0.0974 | T | 0.990 |
| rs11668344 | A | 0.64 | 0.4161 | G | 0.968 |
| rs12294104 | T | 0.20 | 0.2261 | C | 0.974 |
| rs12461110 | G | 0.65 | 0.1193 | A | 0.990 |
| rs1635501 | T | 0.53 | 0.1132 | C | 0.714 |
| rs16991615 | A | 0.07 | 0.9169 | G | 0.993 |
| rs2153157 | A | 0.51 | 0.1207 | G | 0.961 |
| rs2277339 | T | 0.89 | 0.3386 | G | 0.890 |
| rs2303369 | C | 0.57 | 0.1853 | T | 0.993 |
| rs2307449 | T | 0.61 | 0.2188 | G | 0.982 |
| rs2517388 | G | 0.21 | 0.2472 | T | 0.755 |
| rs365132 | T | 0.51 | 0.2997 | G | 0.986 |
| rs4246511 | T | 0.26 | 0.1403 | C | 0.869 |
| rs4693089 | G | 0.48 | 0.2653 | A | 0.889 |
| rs4886238 | A | 0.35 | 0.1613 | G | 0.988 |
|  |  |  |  |  |  |
| **Breast Cancer^e^** |  |  |  |  |  |
| rs1045485 | G | 0.87 | NA | C | 0.996 |
| rs11249433 | G | 0.39 | NA | A | 0.869 |
| rs13281615 | G | 0.41 | NA | A | 0.996 |
| rs13387042 | A | 0.52 | NA | G | 0.994 |
| rs2981582 | A | 0.38 | NA | G | 0.958 |
| rs3803662 | A | 0.27 | NA | G | 1.000 |
| rs3817198 | C | 0.33 | NA | T | 0.974 |
| rs7716600 | A | 0.22 | NA | C | 0.961 |
| rs889312 | C | 0.28 | NA | A | 0.987 |
| rs999737 | C | 0.76 | NA | T | 0.986 |

^a^, risk allele was defined as an allele that was associated with a younger age at menarche, an older age at natural menopause, or an increased risk of breast cancer; ^b^, weight is the beta estimated from linear regression in replication studies within the ReproGen Consortium [[1](#_ENREF_1), [2](#_ENREF_2)], corresponding to the mean change in age at menarche or age at natural menopause per risk allele; ^c^, average observed divided by expected variance for imputed allele dosage. SNPs were either genotyped or imputed in each study based on different genotyping platforms used [[1](#_ENREF_1), [2](#_ENREF_2)]; ^d^, candidate SNPs for age at menarche or age at natural menopause were identified in the ReproGen GWAS meta-analyses [[1](#_ENREF_1), [2](#_ENREF_2)]; ^e^, candidate SNPs for breast cancer were from *Wacholder et al. (N Engl J Med, 2010)*[[3](#_ENREF_3)].

References:

1. Elks CE, Perry JR, Sulem P, Chasman DI, Franceschini N, He C, Lunetta KL, Visser JA, Byrne EM, Cousminer DL, Gudbjartsson DF, Esko T, Feenstra B, Hottenga JJ, Koller DL, Kutalik Z, Lin P, Mangino M, Marongiu M, McArdle PF, Smith AV, Stolk L, van Wingerden SH, Zhao JH, Albrecht E, Corre T, Ingelsson E, Hayward C, Magnusson PK, Smith EN *et al*: **Thirty new loci for age at menarche identified by a meta-analysis of genome-wide association studies**. *Nat Genet* 2010, **42**:1077-1085.

2. Stolk L, Perry JR, Chasman DI, He C, Mangino M, Sulem P, Barbalic M, Broer L, Byrne EM, Ernst F, Esko T, Franceschini N, Gudbjartsson DF, Hottenga JJ, Kraft P, McArdle PF, Porcu E, Shin SY, Smith AV, van Wingerden S, Zhai G, Zhuang WV, Albrecht E, Alizadeh BZ, Aspelund T, Bandinelli S, Lauc LB, Beckmann JS, Boban M, Boerwinkle E *et al*: **Meta-analyses identify 13 loci associated with age at menopause and highlight DNA repair and immune pathways**. *Nat Genet* 2012.

3. Wacholder S, Hartge P, Prentice R, Garcia-Closas M, Feigelson HS, Diver WR, Thun MJ, Cox DG, Hankinson SE, Kraft P, Rosner B, Berg CD, Brinton LA, Lissowska J, Sherman ME, Chlebowski R, Kooperberg C, Jackson RD, Buckman DW, Hui P, Pfeiffer R, Jacobs KB, Thomas GD, Hoover RN, Gail MH, Chanock SJ, Hunter DJ: **Performance of common genetic variants in breast-cancer risk models**. *N Engl J Med* 2010, **362**:986-993.
