# Supplementary material for: Reproductive aging-associated common genetic variants and the risk of breast cancer
Source: Breast Cancer Res. 2012 Mar 20;14(2):R54. doi: 10.1186/bcr3155 (PMC3446388; doi:10.1186/bcr3155)
Supplement: Additional file 3 — Table S2: Characteristics of non-genetic risk factors for breast cancer in each participating study. [file bcr3155-S3.DOCX]

**Table S2. Characteristics of non-genetic risk factors for breast cancer in each participating study.**

|  | **NHS** | | | |  | **WGHS** | | | |  | **SardBC** | | | |
| --- | --- | --- | --- | --- | --- | --- | --- | --- | --- | --- | --- | --- | --- | --- |
| **Characteristics** | **All Subjects** | **Cases** | **Controls** | **Pval** |  | **All Subjects** | **Cases** | **Controls** | **Pval** |  | **All Subjects** | **Cases** | **Controls** | **Pval** |
| Age, yr | 58.4(6.45) | 58.3(6.47) | 58.4(6.44) | 0.85 |  | 54.7(7.12) | 56.2(7.35) | 54.6(7.09) | 3.8E-11 |  | 49.3(14.29) | 57.4(11.56) | 39.7(10.84) | 4.6E-157 |
| Age group |  |  |  |  |  |  |  |  |  |  |  |  |  |  |
| <50 yr | 258(11.3) | 128(11.2) | 130(11.4) | 0.57 |  | 7443(32.0) | 256(23.9) | 7187(32.4) | 1.9E-09 |  | 762(51.4) | 215(26.6) | 547(81.2) | 1.5E-104 |
| 50-59 y | 944(41.3) | 473(41.3) | 471(41.2) |  |  | 10674(45.9) | 511(47.7) | 10163(45.8) |  |  | 351(23.6) | 245(30.3) | 106(15.7) |  |
| 60-69y | 1083(47.4) | 542(47.3) | 541(47.4) |  |  | 4385(18.8) | 256(23.9) | 4129(18.6) |  |  | 228(15.4) | 209(25.8) | 19(2.8) |  |
| ≥70y | 2(0.09) | 2(0.2) | 0(0) |  |  | 774( 3.3) | 48( 4.5) | 726( 3.3) |  |  | 142(9.6) | 140(17.3) | 2(0.3) |  |
| Age at menarche, yr | 12.5(1.39) | 12.5(1.40) | 12.6(1.38) | 0.083 |  | 12.4(1.44) | 12.4(1.45) | 12.4(1.44) | 0.31 |  | - | - | - | - |
| Age at menarche group |  |  |  |  |  |  |  |  |  |  |  |  |  |  |
| ≥14 yr | 464(20.4) | 222(19.6) | 242(21.3) | 0.33 |  | 4243(18.2) | 193(18.0) | 4050(18.3) | 0.53 |  | - | - | - | - |
| 12-14 yr | 1292(56.9) | 641(56.6) | 651(57.3) |  |  | 13363(57.5) | 602(56.3) | 12761(57.5) |  |  | - | - | - | - |
| <12 yr | 514(22.6) | 270(23.8) | 244(21.5) |  |  | 5644(24.3) | 275(25.7) | 5369(24.2) |  |  | - | - | - | - |
| Age at natural menopause^a^, yr | 50.8(3.12) | 50.8(3.21) | 50.7(3.03) | 0.87 |  | 50.6(3.65) | 50.6(3.31) | 50.6(3.66) | 0.79 |  | - | - | - | - |
| Age at natural menopause^a^ group |  |  |  |  |  |  |  |  |  |  |  |  |  |  |
| ≥55 yr | 146(10.8) | 70(10.1) | 76(11.7) | 0.29 |  | 1632(13.6) | 69(11.4) | 1563(13.7) | 7.6E-03 |  | - | - | - | - |
| 45-55 yr | 1159(86.0) | 609(87.4) | 550(84.6) |  |  | 9599(80.2) | 511(84.7) | 9088(79.9) |  |  | - | - | - | - |
| <45 yr | 42(3.1) | 18(2.6) | 24(3.7) |  |  | 744( 6.2) | 23( 3.8) | 721( 6.3) |  |  | - | - | - | - |
| Age at first live birth |  |  |  |  |  |  |  |  |  |  |  |  |  |  |
| <20 yr | 9(0.4) | 3(0.3) | 6(0.5) | 0.092 |  | 1928(9.3) | 57(6.1) | 1871(9.4) | 5.6E-06 |  | - | - | - | - |
| 20-24 yr | 1032(45.6) | 488(42.3) | 544(48.0) |  |  | 10458(50.4) | 440(46.9) | 10018(50.6) |  |  | - | - | - | - |
| 25-29 yr or no birth | 1004(44.4) | 523(46.4) | 481(42.5) |  |  | 6112(29.5) | 309(32.9) | 5803(29.3) |  |  | - | - | - | - |
| ≥30y | 216(9.6) | 114(10.1) | 102(9.0) |  |  | 2240(10.8) | 132(14.1) | 2108(10.6) |  |  | - | - | - | - |
| Family history of breast cancer in first-degree relatives with Breast cancer(yes/no) | 478(20.9) | 274(23.9) | 204(17.9) | 0.00036 |  | 2622(11.5) | 161(15.5) | 2461(11.3) | 5.2E-05 |  | - | - | - | - |
| Parity | 478(20.9) | 274(23.9) | 204(17.9) | 0.00036 |  |  |  |  |  |  |  |  |  |  |
| 0 | 147(6.5) | 90(8.0) | 57(5.0) | 0.017 |  | 2963(12.8) | 154(14.4) | 2809(12.7) | 0.15 |  | - | - | - | - |
| 1-2 | 724(32.0) | 354(31.4) | 370(32.7) |  |  | 8917(38.4) | 417(39.1) | 8500(38.4) |  |  | - | - | - | - |
| ≥ 3 | 1390(61.5) | 684(60.6) | 706(62.3) |  |  | 11316(48.8) | 496(46.5) | 10820(48.9) |  |  | - | - | - | - |
| Menopausal hormone therapy(ever/never) | 1358(81.6) | 685(82.0) | 673(81.1) | 0.62 |  | 10191(43.9) | 508(47.5) | 9683(43.7) | 0.02 |  | - | - | - | - |
| OC use(ever/never) | 1047(45.8) | 547(47.8) | 500(43.8) | 0.056 |  | 16182(69.8) | 694(65.1) | 15488(70.0) | 0.0007 |  | - | - | - | - |
| Adult BMI(kg/m2) | 25.5(4.62) | 25.5(4.58) | 25.4(4.67) | 0.48 |  | 25.3(6.71) | 25.0(5.90) | 25.3(6.74) | 0.14 |  | **-** | - | - | - |

**Table S2. Continued**

|  | **RSI,II** | | | |  | **FHS** | | | |  | **ARIC** | | | |
| --- | --- | --- | --- | --- | --- | --- | --- | --- | --- | --- | --- | --- | --- | --- |
| **Characteristics** | **All Subjects** | **Cases** | **Controls** | **Pval** |  | **All Subjects** | **Cases** | **Controls** | **Pval** |  | **All Subjects** | **Cases** | **Controls** | **Pval** |
| Age, yr | 68.7(9.3) | 66.2 7.3) | 68.8(9.4) | <0.001 |  | 53.2(17.20) | 66.9(14.01) | 52.4(17.03) | <.0001 |  | 54.2(5.59) | 55.3(5.75) | 54.1(5.56) | 0.0047 |
| Age group |  |  |  |  |  |  |  |  |  |  |  |  |  |  |
| <50 yr | 0(0) | 0(0) | 0(0) | 0.016 |  | 1845(47.3) | 25(12.1) | 1820(49.2) | <.0001 |  | 600(25.0) | 44(21.3) | 556(25.3) | 0.0044 |
| 50-59 y | 946(21.1) | 53(24.5) | 893(21.0) |  |  | 840(21.5) | 52(25.1) | 788(21.3) |  |  | 1255(52.3) | 97(46.9) | 1158(52.8) |  |
| 60-69y | 1702(38.0) | 95(44.0) | 1607(37.7) |  |  | 467(12.0) | 40(19.3) | 427(11.6) |  |  | 546(22.7) | 66(31.9) | 480(21.9) |  |
| ≥70y | 1829(40.9) | 68(31.5) | 1761(41.3) |  |  | 753(19.3) | 90(43.5) | 663(17.9) |  |  | 0(0) | 0(0) | 0(0) |  |
| Age at menarche, yr | 13.5(1.64) | 13.4(1.59) | 13.5(1.64) | 0.4 |  | 12.8(1.56) | 12.8(1.63) | 12.8(1.56) | 0.92 |  | 12.9(1.53) | 13.0(1.47) | 12.9(1.53) | 0.55 |
| Age at menarche group |  |  |  |  |  |  |  |  |  |  |  |  |  |  |
| ≥14 yr | 1113(25.9) | 44(21.2) | 1069(26.2) | 0.12 |  | 940(24.1) | 39(27.5) | 901(28.2) | 0.04 |  | 728(30.3) | 66(31.9) | 662(30.2) | 0.66 |
| 12-14 yr | 1925(44.8) | 107(51.4) | 1818(44.5) |  |  | 1816(46.5) | 77(54.2) | 1739(54.5) |  |  | 1310(54.6) | 114(55.1) | 1196(54.5) |  |
| <12 yr | 1256(29.3) | 57(27.4) | 1199(29.3) |  |  | 1149(29.4) | 26(18.3) | 553(17.3) |  |  | 363(15.1) | 27(13.0) | 336(15.3) |  |
| Age at natural menopause^a^, yr | 50.0(3.92) | 50.5(3.92) | 46.0(3.92) | 0.13 |  | 49.69(3.93) | 50.40(3.20) | 49.62(3.99) | 0.036 |  | 48.9(3.81) | 49.4(3.77) | 48.9(3.81) | 0.064 |
| Age at natural menopause^a^ group |  |  |  |  |  |  |  |  |  |  |  |  |  |  |
| ≥55 yr | 328(11.5) | 18(12.2) | 310(11.4) | 0.71 |  | 112(8.12) | 10(8.2) | 102(8.1) | 0.94 |  | 156(6.5) | 15(7.3) | 141(6.4) | 0.78 |
| 45-55 yr | 2117(74.0) | 111(75.5) | 2006(73.9) |  |  | 1154(83.7) | 103(84.4) | 1051(83.6) |  |  | 1960(81.6) | 170(82.1) | 1790(81.6) |  |
| <45 yr | 416(9.3) | 18(12.2) | 398(14.7) |  |  | 113(8.2) | 9(7.4) | 104(8.3) |  |  | 285(11.9) | 22(10.6) | 263(12.0) |  |
| Age at first live birth |  |  |  |  |  |  |  |  |  |  |  |  |  |  |
| <20 yr | 271(6.1) | 12(5.6) | 259(6.1) | 0.83 |  | - | - | - | - |  | - | - | - | - |
| 20-24 yr | 1282(28.6) | 60(27.8) | 1222(28.7) |  |  | - | - | - | - |  | - | - | - | - |
| 25-29 yr or no birth | 2331(52.1) | 111(51.4) | 2220(52.1) |  |  | - | - | - | - |  | - | - | - | - |
| ≥30y | 593(13.2) | 33(15.3) | 560(13.1) |  |  | - | - | - | - |  | - | - | - | - |
| Family history of breast cancer in first-degree relatives with Breast cancer(yes/no) | 121(10.7) | 3(8.6) | 118(10.7) | 0.68 |  | - | - | - | - |  | - | - | - | - |
| Parity |  |  |  |  |  |  |  |  |  |  |  |  |  |  |
| 0 | 669(14.9) | 39(18.1) | 630(14.8) | 0.26 |  | 926(24.4) | 28(14.1) | 898(25.0) | 0.001 |  | 32(1.4) | 2(1.1) | 30(1.5) | 0.40 |
| 1-2 | 2075(46.3) | 105(48.6) | 1970(46.2) |  |  | 1613(42.6) | 87(43.9) | 1526(42.5) |  |  | 939(42.1) | 89(46.6) | 850(41.7) |  |
| ≥ 3 | 1485(33.2) | 63(29.2) | 1422(33.4) |  |  | 1252(33.0) | 83(41.9) | 1169(32.5) |  |  | 1258(56.4) | 100(52.4) | 1158(56.8) |  |
| Menopausal hormone therapy (ever/never) | 863(19.4) | 41(19.0) | 822(19.5) | 0.76 |  | - | - | - | - |  | 570(24.3) | 54(26.3) | 516(24.1) | <0.0001 |
| OC use(ever/never) | 1402(32.0) | 66(31.0) | 1336(32.0) | 0.86 |  | - | - | - | - |  | 1120(46.7) | 98(47.3) | 1022(46.6) | 0.001 |
| Adult BMI(kg/m2) | 27.0(4.18) | 27.5(4.17) | 27.0(4.18) | 0.063 |  | 26.6(5.90) | 27.5(5.36) | 26.5(5.93) | 0.025 |  | 26.5(5.41) | 27.4(5.56) | 26.4(5.39) | 0.014 |

Abbreviations: OC, oral contraceptive; BMI, body mass index. ^a^, based on a subset of women in some studies who were postmenopausal by the time this study was conducted.

Data are presented as the mean (standard deviation) for continuous variables and N (proportion) for categorical variables.
